# Individual-level behavioural smoking cessation interventions tailored for disadvantaged socioeconomic position: a systematic review and meta-regression

**DOI:** 10.1016/S2468-2667(19)30220-8

**Published:** 2019-12-04

**Authors:** Loren Kock, Jamie Brown, Rosemary Hiscock, Harry Tattan-Birch, Charlie Smith, Lion Shahab

**Affiliations:** a Department of Behavioural Science and Health, Institute of Epidemiology and Health Care, University College London, London, UK; b Department for Health, University of Bath, Bath, UK

## Abstract

**Background:**

Socioeconomic inequalities in smoking cessation have led to development of interventions that are specifically tailored for smokers from disadvantaged groups. We aimed to assess whether the effectiveness of interventions for disadvantaged groups is moderated by tailoring for socioeconomic position.

**Methods:**

For this systematic review and meta-regression, we searched MEDLINE, PsycINFO, Embase, Cochrane Central Register, and Tobacco Addiction Register of Clinical Trials and the IC-SMOKE database from their inception until Aug 18, 2019, for randomised controlled trials of socioeconomic-position-tailored or non-socioeconomic-position-tailored individual-level behavioural interventions for smoking cessation at 6 months or longer of follow-up in disadvantaged groups. Studies measured socioeconomic position via income, eligibility for government financial assistance, occupation, and housing. Studies were excluded if they were delivered at the community or population level, did not report differential effects by socioeconomic position, did not report smoking cessation outcomes from 6 months or longer after the start of the intervention, were delivered at a group level, or provided pharmacotherapy with standard behavioural support compared with behavioural support alone. Individual patient-level data were extracted from published reports and from contacting study authors. Random-effects meta-analyses and mixed-effects meta-regression analyses were done to assess associations between tailoring of the intervention and effectiveness. Meta-analysis outcomes were summarised as risk ratios (RR). Certainty of evidence was assessed within each study using the Cochrane risk-of-bias tool version 2 and the grading of recommendations assessment, development, and evaluation approach. The study is registered with PROSPERO, CRD42018103008.

**Findings:**

Of 2376 studies identified by our literature search, 348 full-text articles were retrieved and screened for eligibility. Of these, 42 studies (26 168 participants) were included in the systematic review. 30 (71%) of 42 studies were done in the USA, three (7%) were done in the UK, two (5%) each in the Netherlands and Australia, and one (2%) each in Switzerland, Sweden, Turkey, India, and China. 26 (62%) of 42 studies were trials of socioeconomic-position-tailored interventions and 16 (38%) were non-socioeconomic-position-tailored interventions. 17 (65%) of 26 socioeconomic-position-tailored interventions were in-person or telephone-delivered behavioural interventions, four (15%) were digital interventions, three (12%) involved financial incentives, and two (8%) were brief interventions. Individuals who participated in an intervention, irrespective of tailoring, were significantly more likely to quit smoking than were control participants (RR 1·56, 95% CI 1·39–1·75; I^2^=54·5%). Socioeconomic-position-tailored interventions did not yield better outcomes compared with non-socioeconomic-position-tailored interventions for disadvantaged groups (adjusted RR 1·01, 95% CI 0·81–1·27; β=0·011, SE=0·11; p=0·93). We observed similar effect sizes in separate meta-analyses of non-socioeconomic-position-tailored interventions using trial data from participants with high socioeconomic position (RR 2·00, 95% CI 1·36–2·93; I^2^=82·7%) and participants with low socioeconomic position (1·94, 1·31–2·86; I^2^=76·6%), although certainty of evidence from these studies was graded as low.

**Interpretation:**

We found evidence that individual-level interventions can assist disadvantaged smokers with quitting, but there were no large moderating effects of tailoring for disadvantaged smokers. Improvements in tailored intervention development might be necessary to achieve equity-positive smoking cessation outcomes.

**Funding:**

Cancer Research UK.

## Introduction

In most high-income countries, tobacco smoking prevalence and the associated burden of mortality and disease^1^ are greater in groups with lower socioeconomic position.^2^ Socioeconomic position refers to the social and economic circumstances that influence how different people are positioned within the structure of society.^3^ In England, for example, smoking prevalence is 22·8% among those with manual occupations compared with 12·7% among those with professional to clerical occupations.^4^ These results are supported by observations according to relative socioeconomic position in other high-income, middle-income, and low-income settings.^5^, ^6^, ^7^

**Research in context**

**Evidence before this study**

We searched MEDLINE, PsycINFO, Embase, Cochrane Central Register, and Tobacco Addiction Register of Clinical Trials and the IC-SMOKE project database for studies published in English from database inception until Aug 18, 2019, with the following search terms: smoking cessation or smok* quit* or smok* stop* or smok* cease or smok* cessat* or smok* give up (title and abstract); systematic review or review or RCT or randomi?ed controlled trial or trial or randomi?ed or pragmatic clinical trial (title and abstract); behavio* or behavio?ral support or intervention or counsel* or brief or support or psychol* or individual* or individual-level or behavio?r therapy or cognitive therapy or target* or adapt* or tailor*) not pharma* (title and abstract); and equity or equity impact or inequalit* or poor or disparit* or SES or socio-economic or socio-economic or depriv* or disadvant* social class or occupation or employ or unemploy* or educat* or income or poverty. Tobacco control experts from the authors’ institution and others working within the UK Centre for Tobacco and Alcohol Studies were consulted about relevant submitted or in press articles. Several Cochrane reviews focused on individual-level interventions that were not tailored for low socioeconomic position, including motivational interviewing, behavioural support, and different uses of pharmacotherapy. Bauld and colleagues (2010) examined the equity effect of non-socioeconomic-position-tailored interventions. Reviews by Murray and colleagues (2009) and Bryant and colleagues (2011) focused on interventions targeted at disadvantaged smokers. These reviews suggested that, despite behavioural interventions showing promise for reducing inequalities, smoking cessation generally remains lower among disadvantaged groups. However, these reviews did not examine whether socioeconomic position tailoring moderated intervention effectiveness compared with non-socioeconomic-position-tailored approaches.

**Added value of this study**

To our knowledge, no previous reviews have extended examination of the overall effect of all types of individual-level interventions for smoking cessation in socioeconomically disadvantaged groups to also consider whether socioeconomic position tailoring moderates this effectiveness. We found that both socioeconomic-position-tailored and non-socioeconomic-position-tailored individual-level interventions were effective for smoking cessation in disadvantaged groups. However, there were no large moderating effects of tailoring the interventions for disadvantaged groups compared with not tailoring the interventions. This analysis is an important step forward in gathering evidence about the effectiveness of tailored approaches and encourages further research to improve the effectiveness of equity-focused smoking cessation programmes.

**Implications of all the available evidence**

This systematic review and meta-regression highlights the challenges in achieving improved long-term smoking cessation in disadvantaged groups through tailoring of interventions. Our results do not imply that socioeconomic-position-tailored approaches should be abandoned, but rather that to improve rates of smoking cessation among disadvantaged smokers new, multifaceted approaches are required at the individual, community, and population level, recognising the wider context of socioeconomically disadvantaged smokers. Further research should assess whether current interventions could be further adapted and improved to extend the benefits into longer-term success over and above the effectiveness of non-socioeconomic-position-tailored approaches.

Regular smoking is established and maintained by a variety of molecular and behavioural factors linked to the rapid release of nicotine from cigarettes.^8^, ^9^ Along with other WHO Framework Convention on Tobacco Control measures,^10^ individual-level interventions play an important part in disrupting this motivational process^11^ to support a successful quitting attempt.^12^ However, even with the best support, long-term quitting rates remain low.^13^ Interventions that are tailored to smokers from disadvantaged groups stem from the recognition that smokers from disadvantaged groups have greater difficulty in quitting and remaining abstinent^14^ than do those from more affluent groups. Behavioural interventions delivered at the individual level that recognise the wider context of socioeconomically disadvantaged smokers might prove more successful.^15^, ^16^

The terms socioeconomic position and disadvantaged were operationalised in this Article as populations facing inequalities, marginalisation, or disadvantage in terms of social class, occupation, unemployment, income, poverty, or residential neighbourhood.^15^ In many contexts, ethnicity can change the probability of being socioeconomically disadvantaged.^17^ Some socioeconomic-position-tailored interventions might be delivered to mostly ethnic minority participants—for example, the African American community in the USA. However, given the variety of ethnic distributions and degrees of stigmatisation and the fact that tailoring usually involves some additional cultural adaptation, including such studies was beyond the scope of this Article.

In theory, tailoring interventions to participant characteristics can enhance effectiveness by relating to a participant's life and needs or overcoming specific obstacles to achieve a desired change.^18^ In this Article, we assessed interventions according to whether or not they were tailored to socioeconomic position. Socioeconomic-position-tailored interventions are developed specifically for individuals from socioeconomically disadvantaged groups and aim to overcome some of the specific barriers to quitting that smokers from these groups face, such as financial stress, absence of social support, addiction, insufficient self-efficacy, stress, scarce life opportunities, and little interest in and understanding of tobacco harms.^2^ By contrast, non-socioeconomic-position-tailored interventions are not designed specifically for disadvantaged groups.^19^ In some instances, non-socioeconomic-position-tailored interventions are delivered in a disadvantaged context where recipients have low socioeconomic position, but this does not constitute socioeconomic position tailoring because the intervention has not been developed specifically for such recipients.

Previous reviews have examined the equity effect of non-socioeconomic-position-tailored interventions^20^ or focused on interventions targeted towards disadvantaged smokers.^21^, ^22^ These reviews suggest that despite behavioural interventions showing promise for reducing inequalities, smoking cessation prevalence generally remains lower among disadvantaged groups.^22^, ^23^ A review of research outputs concluded that current research was insufficient to encourage equity-positive improvements in smoking cessation.^24^ To our knowledge, no previous reviews have extended examination of the overall effect of all types of individual-level interventions for smoking cessation in socioeconomically disadvantaged groups to also investigate whether socioeconomic position tailoring moderates this effectiveness.

If socioeconomic-position-tailored interventions are not markedly more effective than non-socioeconomic-position-tailored interventions at increasing smoking cessation among smokers with disadvantaged socioeconomic position then these approaches will require redesign. Therefore, we aimed to assess whether the effectiveness of individual-level smoking cessation interventions for disadvantaged groups was moderated by socioeconomic position tailoring.

## Methods

### Search strategy and selection criteria

This systematic review and meta-regression followed PRISMA guidelines.^25^ We searched MEDLINE, PsycINFO, Embase, Cochrane Central Register, and Tobacco Addiction Register of Clinical Trials and the IC-SMOKE database^26^ from their inception until Aug 18, 2019, for randomised controlled trials,^27^ published in English, of socioeconomic-position-tailored and non-socioeconomic-position-tailored individual-level behavioural interventions for smoking cessation in disadvantaged groups. The following search terms were used: smoking cessation or smok* quit* or smok* stop* or smok* cease or smok* cessat* or smok* give up (title and abstract); RCT or randomi?ed controlled trial or trial or randomi?ed or controlled clinical trial or pragmatic clinical trial (title and abstract); behavio* or behavio?ral support or intervention or counsel* or brief or support or psychol* or individual* or individual-level or behavio?r therapy or cognitive therapy or target* or adapt* or tailor*) not pharma* (title and abstract); and equity or equity impact or inequalit* or under-served or under served or underserved or marginali?ed or poor or affluent or disparit* or SES or socio-economic or socio-economic or depriv* or disadvant* social class or occupation or employ or unemploy* or educat* or income or poverty or neighbo?r* (multiple searches).

This meta-analysis is based on individual participant data. Study authors were contacted if data were not available in a published report. Individual participant-level data were extracted from each study to calculate risk ratios (RRs) and 95% CIs. Studies were excluded if they were delivered at the community or population level, did not report differential effects by socioeconomic position, did not report smoking cessation outcomes from 6 months or longer after the start of the intervention, were delivered at a group level, or provided pharmacotherapy with standard behavioural support compared with behavioural support alone,^28^ because pharmacotherapy itself cannot be tailored to socioeconomic position. However, studies in which pharmacotherapy was given to both the intervention and control groups in addition to a behavioural intervention or control or usual care were included.

LK did the literature search. LK and CS independently screened all abstracts. LK screened all full-text articles and CS screened 10% of full-text articles. Inter-rater reliability at abstract screening (Cohen's κ=0·81) and full study screening (Cohen's κ=0·78) were high. Data were extracted by LK. To check reliability, 10% of data extraction was done independently by HT-B. Percentage agreement was more than 98% after comparison (appendix pp 7–8). Conflicts over inclusion and data extraction were resolved through discussion. LK and HT-B independently assessed the risk of bias and certainty of evidence using the Cochrane risk-of-bias tool version 2 and the GRADE approach^29^ (appendix pp 2–3). The study protocol is available online.

### Data analysis

Duplicate papers reporting data from the same trial were identified and the secondary papers were excluded before data extraction. We extracted data on study type and setting, participant characteristics, intervention details, and smoking cessation outcomes (both self-reported and biochemically verified using expired carbon monoxide or salivary cotinine)^30^ in a customised data extraction form available online.

Diverse interventions, settings, and participants characterise the field of smoking cessation. We judged it likely that the included studies would show heterogeneity in treatment effect (the observed intervention effects being more different from each other than one would expect because of random error alone). As such, the assumptions of a fixed-effect meta-analysis (that all studies in the meta-analysis share a common overall effect size and that all factors that could influence the effect size are the same across studies),^31^ were unlikely to hold. Each study included in this review provides information about a different effect size for smoking cessation. In a random-effects model, the aim is to estimate the mean of a distribution of effects without being overly influenced by any individual study.^32^ Therefore, each study is weighted by the inverse of both its within-study and between-study variance (appendix pp 3–4). The SE of the summary effect is calculated as the square root of this variance.

In random-effects meta-analysis models (restricted maximum-likelihood method),^33^ we calculated pooled RRs with 95% CIs for both socioeconomic-position-tailored and non-socioeconomic-position-tailored interventions as the weighted average of each individual study's estimated intervention effect. All computations were done on a log scale with the log RR, its variance, and SE, before exponentiating the summary effect for interpretation.

We explored heterogeneity by observation of forest plots and use of the χ^2^ test to show whether observed differences in results were compatible with chance alone. We calculated *I*^2^ statistics to examine the level of inconsistency across study findings.^32^ *I*^2^ values reflect the degree of overlap of CIs, with lower values indicating that any observed variance is spurious and higher values suggesting that there are real differences in effect size between studies. Publication bias was assessed using funnel plots. Where visual inspection indicated potential funnel plot asymmetry, we did Egger's regression test to investigate this.^26^ Our analysis followed an intention-to-treat protocol, whereby participants lost to follow-up were classified as continuing to smoke.

We made the following comparisons using forest plots: individual-level interventions (tailored and not tailored to socioeconomic position) versus passive or active control or usual care; socioeconomic-position-tailored individual-level interventions versus passive or active control or usual care; and non-socioeconomic-position-tailored individual-level interventions (subgroups of low socioeconomic position and high socioeconomic position participants) versus passive or active control or usual care.

A conventional meta-analysis attempts to combine results from studies to elucidate a single summary effect size, but diversity in populations and methods among studies often leads to statistical heterogeneity in the true effects of these studies. Meta-regression acts to extend subgroup analyses and allows, in principle, the effects of multiple factors to be investigated simultaneously. Therefore, in contrast to a meta-analysis, meta-regression aims to relate the size of effect to one or more characteristics of the studies involved. In meta-regression, a pooled effect estimate is predicted based on the values of one or more explanatory study-level variables that might influence the size of the intervention effect.^34^ Given a sufficient number of trials (ten studies for each covariate can be sufficient),^34^ we used unadjusted and adjusted mixed-effects meta-regression analyses to assess whether variation among studies in smoking cessation effect size was moderated by tailoring of the intervention for disadvantaged groups. The resulting regression coefficient indicates how the outcome variable (log RR for smoking cessation) changes when interventions take a socioeconomic-position-tailored versus non-socioeconomic-tailored approach. A statistically significant (p<0·05) coefficient indicates that there is a linear association between the effect estimate for smoking cessation and the explanatory variable. More moderators (study-level variables) can be included in the model, which might account for part of the heterogeneity in the true effects. We pre-planned an adjusted model to include important study covariates related to the intensity and delivery of the intervention (number of sessions delivered (above median *vs* below median), whether interventions involved a trained smoking cessation specialist (yes *vs* no), and use of pharmacotherapy in the intervention group (yes *vs* no). These covariates were included a priori as potential confounders given that programmes tailored to socioeconomic position might include more intervention sessions or components or be delivered by different professionals with varying experience. The regression coefficient estimates how the intervention effect in the socioeconomic-position-tailored subgroup differs from the reference group of non-socioeconomic-position-tailored interventions. The true effect for smoking cessation (θi) in the adjusted meta-regression is given by

$$\begin{array}{c} \theta \text{i}=\beta_{0}+\beta_{1}\text{SEP}-{\text{tailored}}_{i}+\beta_{2}{\text{SCS}}_{i}+\beta_{3}{\text{pharmacotherapy}}_{i}\\ +\beta_{4}\text{number of}{\text{sessions}}_{i}+{ɛ}_{\text{k}}+\zeta_{\text{k}} \end{array}$$

where β are the regression coefficients, SEP is socioeconomic position, SCS is smoking cessation specialist, ɛ_k_ is the sampling error through which the effect size of the study deviates from the true effect, and ζ_k_ indicates that the true effect size of the study is sampled from an overall distribution of effect sizes.

Where a non-significant (p>0·05) association between socioeconomic position tailoring and intervention effectiveness was found, we used sensitivity analyses using Bayes factors to examine whether the association reflected evidence of no effect, evidence of an effect, or whether the data were insensitive to detection of an effect.^35^, ^36^

We calculated further exploratory unadjusted univariate and adjusted models to explore the extent to which important study characteristics could explain anticipated heterogeneity in the study estimates.

Analyses were done in the RStudio development environment version 1.1.463 using R version 3.5.2 and the metafor package.^37^ Calculation of Bayes factors was done with an online calculator. The study is registered with PROSPERO, CRD42018103008.

### Role of the funding source

The funder of the study had no role in study design, data collection, data analysis, data interpretation, or writing of the report. The corresponding author had full access to all the data in the study and had final responsibility for the decision to submit for publication.

## Results

**Figure 1:**
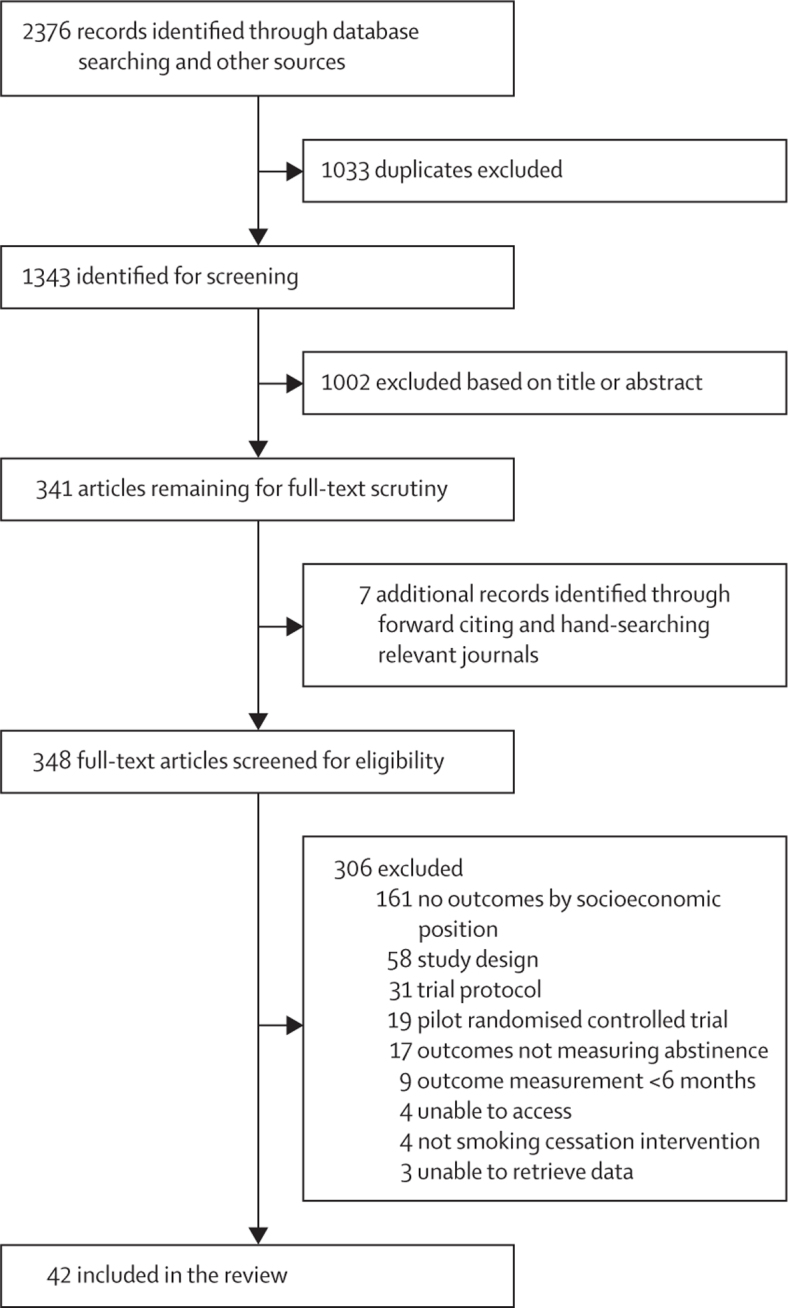
Study selection

Of 2376 studies identified by our literature search, 348 full-text articles were retrieved and screened for eligibility. Of these, 42 studies (26 168 participants) were included in the systematic review (figure 1; table 1). 26 (62%) of 42 studies were trials of socioeconomic-position-tailored interventions and 16 (38) were non-socioeconomic-position-tailored interventions. Measures of socioeconomic position used by studies varied (table 1).

**Table 1:** Study characteristics

	**Country**	**Study design**	**SEP tailoring**	**Sample SEP**	**Women**	**Mean age, years**	**Number randomised**	**Intention to quit**	**Cigarettes per day, mean (95% CI)**	**Intervention condition**	**Control condition**	**Pharmacotherapy**	**Outcome**	**Follow-up**	**Biochemical verification**	**SEP measure**
Abroms at al, 2014^38^	USA	Two-group RCT	No	21·9% high school or lower	66%	38	503	No	17·3 (13·9–20·7)	Text message smoking cessation programme	Link to Smokefree.gov website	None	30-day point prevalence	6 months	Yes	Education
Andrews et al, 2016^39^	USA	Two-group RCT	Yes	79·4% <US$20 000 per year	100%	42	200	Yes	12·7 (7·9–17·6)	Face-to-face individual and group support plus NRT	Written materials	NRT	7-day point prevalence	6 months	Yes	Income
Baker et al, 2018^40^	USA	Two-group RCT	No	Medicaid registered	100%	26	1014	Yes	Not reported	High financial incentive plus counselling	Low financial incentive plus counselling	None	7-day point prevalence	6 months post-birth	Yes	Welfare status
Berndt et al, 2017^41^	Netherlands	Three-group RCT	No	41·8% primary and basic vocational	46%	56	625	No	21·1 (17·8–24·4)	Telephone and face-to-face counselling	Usual care	NRT	12-month continued abstinence	12 months	Yes	Education
Bonevski et al, 2018^42^	Australia	Two-group pragmatic RCT	Yes	94% on state benefits	49%	38	431	No	15 (11·5–18·5)	Brief advice and motivational interviewing	On-screen advice to quit, quitline number	NRT	6-month continued abstinence	6 months	Yes	Welfare status
Brooks et al, 2018^43^	USA	Two-group cluster-randomised trial	Yes	Public housing resident	74%	Not reported	331	Yes	Not reported	Motivational interviewing plus NRT offer	Written materials plus brief advice, NRT offer	NRT offered	7-day and 30-day point prevalence	12 months	Yes	Housing tenure
Brown et al, 2014^44^	UK	Two-group RCT	Yes	46·4% long-term unemployed or routine and manual occupation	63%	39	4613	Yes	18·6 (17·5–19·7)	An interactive website intervention	Static website with brief advice	None	6-month continued abstinence	6 months	Yes	Occupation
Choi et al, 2014^45^	USA	Two-group RCT	Yes	61·1% high school or less	20%	42	145	No	21·0 (14·0–28·0)	Website plus telephone support and NRT	Telephone support and NRT	NRT	7-day point prevalence	6 months	Yes	Education
Curry et al, 2003^46^	USA	Two-group RCT	Yes	43·2% <US$10 000 per year	100%	34	303	No	12·1 (8·26–15·9)	Motivational interviewing plus telephone support	Usual care	None	7-day point prevalence	12 months	No	Income
Davis et al, 2014^47^	USA	Two-group RCT	Yes	49·5% high school or less	50%	42	196	Yes	Not reported	Mindfulness training plus NRT	Telephone support plus NRT	NRT	7-day point prevalence	6 months	Yes	Education
Danan et al, 2018^48^	USA	Two-group RCT	No	49·5% high school or less	5%	60	2430	No	≤10=36%, 11–20=42%, ≥21=22%	Proactive outreach with offer of telephone counselling or referral to in-person counselling	Usual care	NRT, buproprion, or varenicline available	6-month continued abstinence	6 months	No	Education
Etter and Schmid, 2016^49^	Switzerland	Two-group RCT	No	18% unemployed	50%	32	805	Yes	16·0 (13·4–18·6)	Written materials, website access, and escalating financial rewards	Written materials plus website access	None	12-month continued abstinence	6 months	Yes	Occupation
Fraser et al, 2017^50^	USA	Two-group RCT	Yes	Medicaid registered	61%	45	1900	No	17·2 (15·5–18·9)	Telephone support plus extra financial incentive	Telephone support plus financial incentive	None	7-day point prevalence	6 months	Yes	Welfare status
Free et al, 2011^51^	UK	Two-group RCT	No	31% manual occupation	45%	37	5800	Yes	Not reported	Text messaging smoking cessation programme	Text messages unrelated to quitting	None	6-month continued abstinence	6 months	Yes	Occupation
Froelicher et al, 2010^52^	USA	Two-group RCT	Yes	58·3% <US$15 000 per year	73%	47	60	No	11·3 (2·5–20·1)	Face-to-face support plus industry and media messaging	Face-to-face support	Unclear	7-day point prevalence	6 months	Yes	Income
Fu et al, 2016^53^	USA	Two-group RCT	Yes	Medicaid registered	71%	Not reported	2406	No	13·6 (12·2–15·0)	Usual care plus proactive telephone and written outreach and NRT	Usual care	NRT	12-month continued abstinence	12 months	No	Welfare status
Glasgow et al, 2000^54^	USA	Two-group RCT	No	42·7% high school or less	100%	24	1154	No	12·0 (10·1–13·9)	Brief behavioural support and clinician advice	Written materials and advice	None	30-day point prevalence	6 months	Yes	Income
Gordon et al, 2010^55^	USA	Two-group RCT	Yes	At or below 200% of US federal poverty level	58%	41	2637	No	Not reported	Brief advice and assistance and NRT	Usual care	NRT	6-month continued abstinence	7·5 months	No	Income
Haas et al, 2015^56^	USA	Two-group RCT	Yes	62·3% medical or medicare recipient	69%	50	707	No	15·0 (12·3–17·7)	Telephone support plus NRT	Usual care	NRT	7-day point prevalence	9 months	No	Welfare status
Yilmaz et al, 2006^57^	Turkey	Three-group RCT	No	50·5% <US$250 per month	100%	Not reported	363	No	6·30 (3·67–8·94)	General health information, child and mother health risks, and booklet	General health information	None	7-day point prevalence	6 months	No	Income
Kendzor et al, 2012^58^	USA	Two-group RCT	No	61·1% unemployed	52%	42	379	No	Not reported	Standard care plus intervention delivered using palmtop computer	Self-help materials plus counselling and NRT	NRT	30-day point prevalence	6 months	Yes	Employment
Lasser et al, 2017^59^	USA	Two-group RCT	No	55% <US$20 000 per year	54%	50	352	Yes	15 (11·1–18·9)	Enhanced usual care (face-to-face support plus written materials and information on local cessation resources)	Usual care (face-to-face support)	NRT offered	7-day point prevalence	12 months	Yes	Income
Lepore et al, 2018^60^	USA	Two-group RCT	Yes	78·7% income below poverty level	84%	33	327	No	11·5 (7·85–15·1)	Face-to-face and telephone support	Nutrition intervention	None	7-day point prevalence	12 months	Yes	Income
Lou et al, 2013^61^	China	Two-group RCT	No	Mean income $US3015 per year	52%	Not reported	3562	No	Not reported	General practioner face-to-face support	Usual care	None	6-month continued abstinence	30 months	Yes	Income
Marks and Sykes, 2002^62^	UK	Two-group RCT	No	37% unemployed	Not reported	Not reported	260	No	Not reported	Enhanced written materials package	Written materials	None	7-day point prevalence	12 months	Yes	Income
McClure et al, 2018^63^	USA	Two-group RCT	Yes	62·6% <US$20 000 per year	62%	44	718	No	19·1 (16·2–22·0)	Telephone support, written materials, and oral health intervention	Telephone support plus written materials	NRT offered	7-day point prevalence	12 months	No	Income
Mundt et al, 2019^64^	USA	Two-group RCT	Yes	Medicaid registered	60%	45	1900	No	17·2 (15·5–18·9)	Financial incentive for taking offered counselling calls	Offer of counselling calls	Offered	7-day point prevalence	6 months	Yes	Welfare status
Nohlert et al, 2009^65^	Sweden	Two-group RCT	No	23% 0–9 years education	80%	Not reported	300	No	Not reported as mean	Multiple face-to-face support sessions	One face-to-face support session and written materials	None	7-day point prevalence	12 months	No	Education
Okuyemi et al, 2007^66^	USA	Two-group cluster-randomised trial	No	Public housing resident	72%	46	174	No	17·5 (11·6–23·4)	Face-to-face and written materials addressing smoking cessation plus NRT	Face-to-face and written materials addressing nutrition	NRT	7-day point prevalence	6 months	Yes	Housing tenure
Pbert et al, 2004^67^	USA	Two-group cluster-randomised trial	Yes	46·7% less than high school	100%	26	609	No	16·7 (13·6–19·7)	Face-to-face support and written materials	Usual care	None	7-day point prevalence	6 months post-birth	Yes	Income
Prokhorov et al, 2008^68^	USA	Two-group RCT	Yes	Community college students	59%	23	426	No	12·5 (9·2–15·7)	Computer-assisted support and motivational interviewing	Brief face-to-face support and written materials	None	7-day point prevalence	10 months	Yes	Income
Rash et al, 2018^69^	USA	Two-group RCT	Yes	Homeless	26%	45	70	Yes	15·4 (6·2–24·6)	Standard care plus financial incentives	Face-to-face counselling	NRT	7-day point prevalence	6 months	Yes	Housing tenure
Ruger et al, 2008^70^	USA	Two-group RCT	Yes	Medicaid registered	100%	26	302	No	Not reported	Motivational interviewing and relapse prevention support	Usual care	None	30-day point prevalence	6 months post-birth	Yes	Welfare status
Sarkar et al, 2017^71^	India	Two-group cluster-randomised trial	Yes	75·9% <US$70 per month	20%	46	1213	No	Not reported	Brief face-to-face support and breathing exercises	Very brief advice	None	6-month continued abstinence	7 months	Yes	Income
Sheffer et al, 2017^72^	USA	Two-group RCT	Yes	56·8% <US$10 000 per year	19%	48	256	Yes	13·8 (9·4–18·2)	Enhanced standard care: SEP-tailored face-to-face cognitive behavioural treatment for tobacco dependence, NRT	Face-to-face cognitive behavioural treatment for tobacco dependence, NRT	NRT	7-day point prevalence	6 months	Yes	SEP (income and education)
Solomon et al, 2005^73^	USA	Two-group RCT	Yes	Medicaid registered	100%	34	330	Yes	23·6 (18·9–28·3)	Proactive telephone support plus pharmacotherapy	Pharmacotherapy	NRT	7-day and 30-day point prevalence	6 months	No	Welfare status
Solomon et al, 2000^74^	USA	Two-group RCT	Yes	Medicaid registered	100%	33	214	Yes	23·7 (17·7–30·0)	Proactive telephone support plus pharmacotherapy	Pharmacotherapy	NRT	7-day point prevalence	6 months	Yes	Welfare status
Sorensen et al, 2007^75^	USA	Two-group RCT	Yes	Routine and manual occupation	6%	41	674	No	Not reported	Telephone delivered motivational interviewing, tailored written materials, and NRT	Written materials	NRT offered	7-day point prevalence	6 months	No	Occupation
Stanczyk et al, 2016^76^	Netherlands	Three-group RCT	No	33·6% low education	62%	45	2099	Yes	18·9 (17·2–20·6)	Text and internet-based intervention	General advice	None	12-month continued abstinence	12 months	Yes	Education
Stanton et al, 2004^77^	Australia	Two-group RCT	Yes	Undefined lower SEP (public hospital setting)	0	Not reported	561	No	Not reported	Smoking cessation video plus NRT	Written materials	NRT	Not reported	6 months	Yes	Education or occupation
Strecher et al, 2008^78^	USA	Two-group RCT	No	36·2% high school or less	60%	Not reported	1866	Yes	Not reported	High-depth website intervention plus NRT	Low-depth website intervention plus NRT	NRT	7-day point prevalence	6 months	No	Education
Vidrine et al, 2019^79^	USA	Three-group RCT	No	70% high school or less	51%	49	624	Yes	≤10=30%, 11–20=46%, ≥21=24%	NRT plus text and telephone calls	NRT alone	NRT	30-day point prevalence	6 months	Yes	Education

SEP=socioeconomic position. RCT=randomised controlled trial. NRT=nicotine replacement therapy.

30 (71%) of 42 studies were done in the USA,^38–40,43,45–48,50,52–56,58–60,63,64,66–70,72–75,78,79^ three (7%) were done in the UK,^44^, ^51^, ^62^ two (5%) each in the Netherlands^41^, ^76^ and Australia,^42^, ^77^ and one (2%) each in Switzerland,^49^ Sweden,^65^ Turkey,^57^ India,^71^ and China.^61^

Ten studies recruited participants during hospital or clinic visits related to general health, cardiac health, dental health, or the health of a participant's child.^41,46,54,55–57,59,60,65,77^ Nine studies recruited only women.^39^, ^40^, ^46^, ^54^, ^57^, ^67^, ^70^, ^74^, ^79^ Three studies exclusively included pregnant women^40^, ^67^, ^70^ and one study recruited only men whose partners were pregnant.^77^ White participants were the majority in 23 studies^38,40,44,45,47,48,51,53,54,56,62,63,65,67–71,73–77^ and African American participants were the majority in 12 studies.^39^, ^43^, ^46^, ^50^, ^52^, ^55^, ^58^, ^59^, ^60^, ^64^, ^66^, ^79^ One study recruited only Chinese participants,^61^ and another only Indian participants.^71^

In-person or telephone support typically included one or more sessions with a health professional who assisted in the quit attempt. These professionals included clinicians, nurses, or health educators, who either provided smoking cessation support as part of their job or worked as a smoking cessation specialist. Digital behavioural support involved interactive and tailored smoking cessation support delivered via text messages, or on a website or page accessible on a computer or other device. Financial incentive condition participants received incentives that were conditional upon them attending support sessions or health visits or contingent upon biochemically validated smoking abstinence at follow-up. Brief interventions consisted of brief advice and assistance related to smoking cessation and outlined general health risks from smoking.

Overall, six (14%) of 42 included studies were classified as being at low risk of bias on all domains considered in the assessment (appendix pp 2–3).

**Figure 2:**
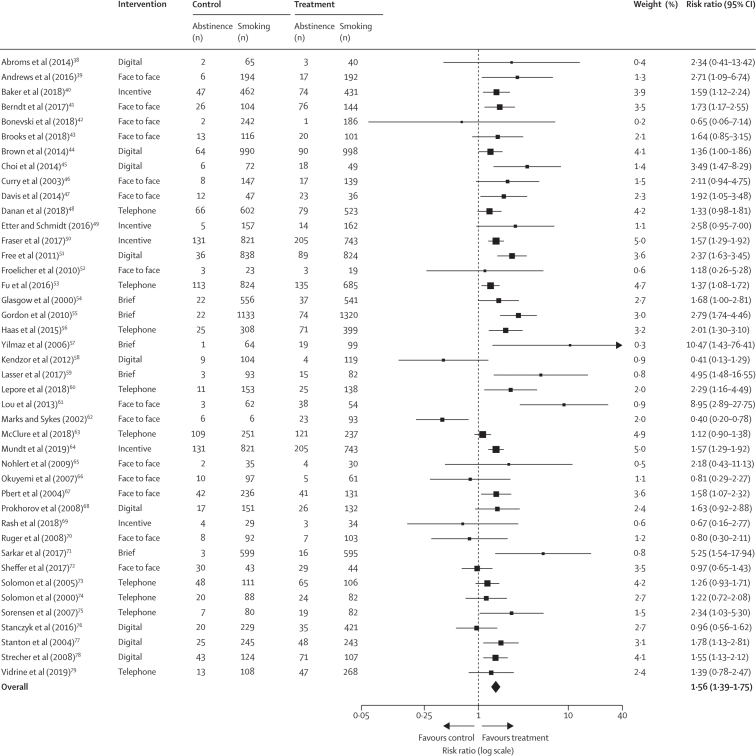
Individual-level interventions compared with control or usual care in socioeconomically disadvantaged groups Outcome was smoking cessation at ≥6 months follow-up.

A pooled effect size was estimated based on the 42 studies of socioeconomic-position-tailored and non-socioeconomic-position-tailored individual-level interventions in groups with low socioeconomic position (figure 2). Individuals with low socioeconomic position who participated in an intervention were significantly more likely to quit smoking than those with low socioeconomic position in control groups (RR 1·56, 95% CI 1·39–1·75). We found evidence of moderate heterogeneity in the effect size between trials (*I*^2^=54·5%). The certainty of evidence for this comparison was deemed to be moderate. A funnel plot suggested that there was no reporting bias for smoking cessation outcomes (appendix p 4).

In an unadjusted univariate model, tailoring of interventions for disadvantaged groups was not associated with smoking cessation effect size (table 2). This absence of association between tailoring of the intervention and intervention effect was also evident in the pre-planned model adjusted for the number of sessions delivered (table 3; model 1), whether interventions were delivered by a smoking cessation specialist and whether the interventions involved the use of pharmacotherapy. However, we found evidence of some intercorrelation among study characteristics in model 1 (table 3), whereby interventions that were delivered by a trained specialist generally involved a greater number of sessions. Therefore, we removed the number of sessions covariate and reran the analyses (table 3; model 2).

**Table 2:** Unadjusted univariate associations between intervention factors and effect size of intervention

	**β (SE)**	Risk ratio (95% CI)*	**p value**	I**^2^**	**Adjusted R^2^**
Tailored for low socioeconomic position†	−0·02 (0·13)	1·02 (0·79–1·32)	0·86	57·02%	0·00%
Trained specialist‡	−0·23 (0·12)	0·79 (0·63–0·99)	0·048	50·38%	13·65%
Pharmacotherapy§	0·27 (0·13)	1·31 (1·01–1·68)	0·045	41·27%	41·20%
Number of sessions¶	−0·01 (0·12)	1·00 (0·78–1·27)	0·99	56·55%	0·00%
Active control‖	−0·03 (0·13)	0·97 (0·75–1·25)	0·80	57·21%	0·00%
Type of support**	−0·26 (0·14)	0·77 (0·58–1·02)	0·064	52·21%	3·48%
Risk of bias††	−0·33 (0·18)	0·72 (0·51–1·02)	0·068	52·66%	5·81%
Biochemical verification‡‡	−0·03 (0·13)	0·97 (0·74–1·26)	0·80	57·39%	0·00%
Intention to quit§§	−0·06 (0·13)	0·94 (0·73–1·20)	0·60	56·28%	0·00%

* Calculated by exponentiating log-transformed estimates of intervention effect.

† Socioeconomic-position-tailored *vs* non-socioeconomic-position-tailored intervention.

‡ Intervention involved provider trained in smoking cessation *vs* not trained in smoking cessation.

§ Pharmacotherapy delivered *vs* not delivered.

¶ Number of sessions delivered in intervention >4 *vs* ≤4.

‖ Active control *vs* inactive control.

** Digital or face-to-face or telephone intervention *vs* other intervention (financial incentives and brief interventions).

†† High or some concerns over risk of bias *vs* low risk of bias.

‡‡ Biochemically verified smoking cessation *vs* no biochemically verified smoking cessation.

§§ Intention to quit *vs* no intention to quit.

**Table 3:** Adjusted associations between tailoring and effect size of intervention

	**β (SE)**	Risk ratio*(95% CI)	**p value**
**Model 1**			
Tailored for low SEP†	−0·01 (0·12)	1·01 (0·80–1·28)	0·93
Trained specialist‡	−0·28 (0·13)	0·76 (0·58–0·98)	0·0035
Pharmacotherapy§	0·24 (0·14)	1·27 (0·96–1·67)	0·089
Number of sessions¶	0·11 (0·13)	1·12 (0·87–1·45)	0·38
**Model 2**			
Tailored for low SEP†	0·01 (0·11)	1·01 (0·81–1·27)	0·93
Trained specialist‡	−0·21 (0·11)	0·81 (0·65–0·99)	0·049
Pharmacotherapy§	0·25 (0·13)	1·29 (0·99–1·67)	0·058

SEP=socioeconomic position.

* Calculated by exponentiating log-transformed estimates of intervention effect. Associations after mutual adjustment for all variables listed in this table.

† SEP-tailored *vs* non-SEP-tailored intervention.

‡ Intervention involved provider trained in smoking cessation *vs* not trained in smoking cessation.

§ Pharmacotherapy delivered *vs* not delivered.

¶ Number of sessions delivered in intervention >4 *vs* ≤4.

Based on an expected RR of 1·5, the calculated Bayes factor for model 2 (0·291) indicated weak evidence that tailoring had no effect on intervention effectiveness. Repeating the calculation based on an expected effect size of 1·1 showed that the data were insensitive to detection of small effects (Bayes factor=0·81).

Exploratory unadjusted univariate models showed no evidence of an association between biochemical verification and smoking cessation effect size, but behavioural support (digital or in-person or telephone), studies with some concerns in at least one domain of the Cochrane risk-of-bias tool for this result, but no high risk of bias for any domain, and pharmacotherapy had meaningful associations with effect size (table 2). An adjusted model including these three variables reduced the heterogeneity in the effect size between trials (*I*^2^=16·55%, R^2^_adjusted_=82·09%; p=0·0027) compared with the result from the primary meta-analysis (*I*^2^=54·50%; appendix p 5).

We estimated a pooled effect size based on the 26 studies of socioeconomic-position-tailored interventions (appendix p 5). Smokers with disadvantaged socioeconomic position who participated in a socioeconomic-position-tailored intervention were significantly more likely to quit smoking than were those in the control group (RR 1·54, 95% CI 1·37–1·72) with some evidence of heterogeneity in the effect size between trials (*I*^2^=38·10%).

**Figure 3:**
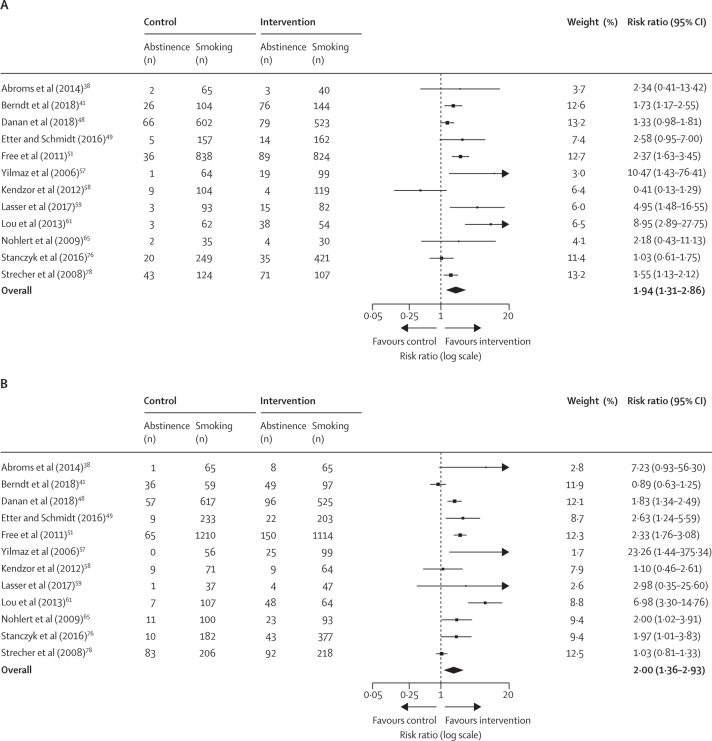
Non-socioeconomic-position-tailored interventions compared with control or usual care in participants with low socioeconomic position (A) and participants with high socioeconomic position (B)

We estimated pooled effect sizes separately for participants with low socioeconomic position and participants with high economic position based on the 12 studies of non-socioeconomic-position-tailored interventions that reported outcomes (figure 3). Four non-socioeconomic-position-tailored interventions were excluded from this comparison as they were delivered in a low socioeconomic position context and did not provide outcome data for participants with high socioeconomic position.

Individuals with low and high socioeconomic position who participated in a non-socioeconomic-position-tailored intervention were significantly more likely to quit smoking than were controls. However, we found evidence of high heterogeneity in the effect size between trials for both the low socioeconomic position and high socioeconomic position subgroups (*I*^2^=76·6% and *I^2^*=82·7%, respectively; figure 3). The results of our subgroup analysis suggest that there were no differences between the estimates of smoking cessation according to the socioeconomic position of participants (appendix pp 5–6).

Funnel plots indicated potential reporting bias due to studies suggesting a beneficial effect being more likely to be published than studies showing no effect (appendix p 6). Egger's test for funnel plot asymmetry showed no difference with respect to the low socioeconomic position participant analysis, but a significant difference for the high socioeconomic position analysis (appendix p 7).

## Discussion

We found consistent evidence that individual-level interventions for smoking cessation in socioeconomically disadvantaged groups are effective for smoking cessation. However, we found no evidence that tailoring interventions for smokers with low socioeconomic position significantly moderated effectiveness compared with non-socioeconomic-position-tailored interventions. Bayes factors indicated that there were no large moderating effects, but that the data were insensitive to detection of smaller moderating effects. This finding was not surprising considering that meta-analyses of non-socioeconomic-position-tailored interventions showed similar effect sizes for smoking cessation in separate models for participants with high socioeconomic position and with low socioeconomic position from the same study. However, the estimates for subgroups should be interpreted with caution given that overall the evidence from these studies was deemed to be of low certainty.

Tailored individual-level approaches are expected to have an important role in reducing health inequalities by addressing some of the needs specific to disadvantaged smokers. However, our results imply that such tailoring has not yet improved effectiveness compared with non-socioeconomic-position-tailored approaches. Nevertheless, such programmes have shown general effectiveness so should not be withdrawn without replacement.^22^, ^80^ To improve the prevalence of smoking cessation among disadvantaged smokers, a new, multifaceted approach is required at the individual, community, and population level. Compared with those with more advantaged socioeconomic position, individuals with low socioeconomic position face more facilitators to smoking uptake and more barriers to quitting,^2^ which might outweigh the benefits of tailoring interventions at the individual level.

Comparing results from separate meta-analyses using data from participants with low socioeconomic position and participants with high socioeconomic position from the same trial showed that the effects of non-socioeconomic-position-tailored interventions on smoking cessation were similar in all participants. This finding contrasts with a previous review,^81^ which suggested that non-socioeconomic-position-tailored smoking cessation support interventions were likely to be equity negative (helping participants with advantaged socioeconomic position to quit more than disadvantaged participants). However, this divergence should be interpreted with caution as the inclusion criteria between the studies differed. The current systematic review only included randomised controlled trials of individual-level interventions measuring smoking cessation at least 6 months after baseline. The previous review^81^ largely focused on face-to-face behavioural support and included observational and correlational designs and randomised controlled trials that involved the use of pharmacotherapy alone. Furthermore, in response to inequalities in access, provision of smoking cessation services in some low-socioeconomic-position areas of the UK has improved, with results from programmes in Scotland indicating improvements in quitting success among disadvantaged smokers.^61^ These data support the finding from the current review that non-socioeconomic-position-tailored interventions appear to have a similar effectiveness for quitting smoking success across the social gradient, if access to such services is provided.

Nine studies included in this review recruited women only, whereas one study recruited men only. This focus might be a response to the evidence of higher smoking prevalence and health inequalities among disadvantaged ethnic minority women^25^ and the potential opportunity for a smoking cessation intervention when women are in the clinic either during or following pregnancy.

30 studies in our review used point prevalence (7-day or 30-day) rather than continued abstinence outcomes. Although there is some debate as to which is a more robust measure, a 2010 systematic review^82^ comparing these two outcome measures concluded that they are highly correlated and produce similar effect sizes for smoking cessation.

This systematic review is not without limitations. Although several covariates were prespecified in the protocol, it was not possible to do the same for all other potentially important covariates and this might result in false-positive conclusions. Therefore, results indicating a reduction in heterogeneity compared with the primary meta-analysis should be viewed as exploratory. Furthermore, study characteristics included in the meta-regression might have been highly correlated, such that an observed association with one study characteristic is in fact reflective of a true association with another correlated characteristic that has not been measured. There was some evidence of clustering of study characteristics, whereby more sessions appeared to take place if a trained specialist was delivering the intervention. It is also possible that the effectiveness of behavioural support depended on the skill of the practitioner delivering it;^83^ unfortunately a variable to assess practitioner skill was not available for most studies analysed, so meaningful adjustment for this was not possible. However, such effects are generally relatively small^83^ and so unlikely to have overly biased our results. Furthermore, since we included study quality (which measures bias in trials) in the meta-regression, we argue that we attempted to account for therapist effects as far as possible given the available information. Our risk of bias assessment included deviations from the intended interventions. In cases in which the original study provided no information for this factor, the potential bias was noted and included in the final assessment for overall risk of bias. Other measures of effectiveness for smoking cessation in interventions tailored for disadvantaged groups, such as time to relapse and abstinence at earlier follow-up timepoints, might provide a more nuanced picture of study results.

There are potential limitations related to the operationalisation of socioeconomic position in this Article. Although 39 (93%) of 42 studies were done in high-income countries, there are often between-country differences in terms of how socioeconomic position is experienced and how this influences health behaviour.^3^ Furthermore, the socioeconomic position of the underlying sample populations in each study might have differed between socioeconomic-position-tailored and non-socioeconomic-position-tailored interventions. Were this true, the apparent effectiveness of non-socioeconomic-position-tailored interventions for smokers with low socioeconomic position discussed in this review might reflect the recruitment of more socioeconomically advantaged participants than in trials of socioeconomic-position-tailored interventions. Trials of non-socioeconomic-position-tailored interventions that report outcomes by socioeconomic position might also differ from non-socioeconomic-position-tailored interventions that do not report this. Such studies might focus more on socioeconomic position issues despite not explicitly reporting on tailoring of the intervention to populations with different socioeconomic positions, which might lead to underestimating the moderating effect of socioeconomic position tailoring. Therefore, future research in this field should consider using a standardised index of socioeconomic position to allow valid comparison between levels of deprivation across populations.

During the study screening process, it became apparent that relevant studies (n=161) might have been excluded because they did not report their outcomes by socioeconomic position, despite potentially having a socioeconomically diverse sample of participants. Given the persistent inequalities in smoking rates worldwide, it is becoming ever more important that smoking cessation trials, where possible, collect and report outcomes by socioeconomic position. Studies are typically not powered for robust subgroup analyses by socioeconomic position, but if outcomes are reported in this way then they can be cumulatively included in pooled effect size estimates in future reviews. The certainty of evidence of studies included in this review was rated as moderate for the primary analysis and low for the secondary analyses. As such, it remains possible that the true effects are different to what was estimated.

Despite these limitations, this study has several strengths. To our knowledge, no previous reviews have examined whether socioeconomic position tailoring moderates the effectiveness of individual-level behavioural smoking cessation interventions at 6 months or later in socioeconomically disadvantaged groups. Inclusion of 42 studies in our systematic review made it possible to do a meta-regression analysis, which is a useful tool to extend the analysis and relate the size of treatment effect in clinically and methodologically diverse studies to relevant study characteristics. Considering the growing number of interventions that involve some form of tailoring for disadvantaged groups, this analysis is an important step towards gathering evidence about their effectiveness and might also encourage further equity-focused research that will improve the effectiveness of smoking cessation programmes. Future research in this area should also consider assessing what the most effective components of socioeconomic-position-tailored interventions are by using an appropriate theory-informed taxonomy.^26^, ^84^

This systematic review and meta-regression highlights that although both socioeconomic-position-tailored and non-socioeconomic-position-tailored individual-level interventions for smoking cessation in socioeconomically disadvantaged groups are effective for smoking cessation, based on the evidence available for this review, there is currently no evidence for large moderating effects of tailoring for disadvantaged smokers.
