# Supplementary material for: Individual-level behavioural smoking cessation interventions tailored for disadvantaged socioeconomic position: a systematic review and meta-regression
Source: Lancet Public Health. 2019 Dec 4;4(12):e628–44. doi: 10.1016/S2468-2667(19)30220-8 (PMC7109520; doi:10.1016/S2468-2667(19)30220-8)
Supplement: Supplementary appendix [file mmc1.pdf]

THE LANCET
Public Health

Supplementary appendix

This appendix formed part of the original submission and has been peer reviewed. We post it as supplied by the authors.

Supplement to: Kock L, Brown J, Hiscock R, Tattan-Birch H, Smith C, Shahab L. Individual-level behavioural smoking cessation interventions tailored for disadvantaged socioeconomic position: a systematic review and meta-regression. *Lancet Public Health* 2019; **4:** e628–44.

Supplementary appendix

GRADE ratings and their interpretation

Symbol	Certainty*	Interpretation
⊕⊕⊕⊕	**High**	We are very confident that the true effect lies close to that of the estimate of the effect.
⊕⊕⊕⊖	**Moderate**	We are moderately confident in the effect estimate: the true effect is likely to be close to the estimate of the effect, but there is a possibility that it is substantially different.
⊕⊕⊖⊖	**Low**	Our confidence in the effect estimate is limited: the true effect may be substantially different from the estimate of the effect.
⊕⊖⊖⊖	**Very low**	We have very little confidence in the effect estimate: the true effect is likely to be substantially different from the estimate of effect.

The GRADE system rates the quality/certainty of evidence for each outcome from a rating of high to very low after considering and making a judgement based 8 assessment criteria (Risk of Bias, Inconsistency, Indirectness, Imprecision, and Publication Bias). Table taken from the GRADE Handbook, available at http://gdt.guidelinedevelopment.org/app/handbook/handbook.html#h.9rdbelsnu4iy

‡Downgraded due to concerns related to blinding, measurement of outcome in some studies and moderate levels of heterogeneity in effect estimate.

§Downgraded due to concerns related to blinding, measurement of outcome in some studies, high levels of heterogeneity in effect estimate and some evidence for potential publication bias.

¶Only 12 out of 16 non SEP-tailored interventions reported outcomes for both high-SEP and low-SEP participants.

Search terms for electronic databases

Smoking cessation interventions: smoking cessation or smok* quit* or smok* stop* or smok* cease or smok* cessat* or smok* give up (title and abstract)

RCT design: RCT or randomi?ed controlled trial or trial or randomi?ed or controlled clinical trial or pragmatic clinical trial (title and abstract)

Individual-level behavioural support: behavio* or behavio?ral support or intervention or counsel* or brief or support or psychol* or individual* or individual-level or behavio?r therapy or cognitive therapy or target* or adapt* or tailor*) not pharma* (title and abstract)

Socio-economic position: equity or equity impact or inequalit* or under-served or under served or underserved or marginali?ed or poor or affluent or disparit* or SES or socio-economic or socio-economic or depriv*OR disadvant* social class or occupation or employ or unemploy* or educat* or income or poverty or neighbo?r* (multiple searches)

Table s1: Methodological quality summary and risk of bias assessment

Study	The randomization process	Deviations from the intended interventions (effect of assignment to intervention)	Deviations from the intended interventions (effect of adhering to intervention)	Missing outcome data	Risk of bias in measurement of the outcome	Selection of the reported result	Overall judgement
Abroms 2014	+	+	+	+	+	+	+
Andrews 2016	+	+	+	+	+	+	+
Baker 2018	+	+	?	?	?	?	?
Berndt 2018	+	?	?	?	?	+	?
Bonevski 2018	?	+	-	+	+	+	-
Brooks 2017	?	-	-	?	+	?	-
Brown 2014	+	+	+	+	+	+	+
Choi 2014	?	+	-	-	-	+	-
Curry 2003	+	?	?	+	+	?	?
Davis 2014	?	?	-	-	+	+	-
Danan 2018	+	+	?	?	-	+	-
Etter 2016	+	+	+	?	+	+	?
Fraser 2017	+	+	?	+	?	?	?
Free 2011	+	+	+	+	+	+	+
Froelicher 2010	+	?	-	-	+	-	-
Fu 2016	+	?	?	?	-	+	?
Glasgow 2000	+	+	?	?	+	+	?
Gordon 2010	?	?	?	-	-	+	-
Haas 2015	?	+	?	+	-	+	-
Karacan 2006	?	?	+	+	-	+	-
Kendzor 2012	+	?	?	+	+	+	?
Lasser 2017	+	+	?	+	+	+	?
Lepore 2018	+	+	+	+	+	+	+
Lou 2013	+	?	?	?	+	+	?
Marks 2002	?	?	?	-	+	+	-
McClure 2018	+	+	+	+	?	+	?
Mundt 2019	+	?	+	?	+	?	?
Nohlert 2009	+	+	?	+	-	+	-
Okuyemi 2007	+	+	?	?	+	+	?
Pbert 2004	?	+	?	?	+	+	?
Prokhorov 2008	+	-	?	?	+	?	-
Rash 2018	+	+	?	+	?	+	?
Ruger 2008	+	?	+	+	+	+	?
Sarkar 2017	+	+	+	+	+	+	+
Sheffer 2017	+	?	+	+	+	+	?
Solomon 2005	?	+	+	+	?	?	?
Solomon 2000	?	+	+	?	?	?	?
Sorensen 2007	+	+	?	?	-	+	-

Stanczyk 2016	+	+	+	?	?	+	?
Stanton 2004	?	+	?	?	?	?	?
Strecher 2008	+	+	+	+	?	+	?
Vidrine 2019	+	+	+	+	?	?	?

*Low risk of bias = “+”; some concerns = “?”; high risk of bias = “-“

Risk of bias summary

Five studies were judged to be at high risk of bias due to potential deviations from the intended interventions in the trial.[48,49,57,86,89] Examples of this include poor participation in or adherence to intervention components (such as behavioural support sessions or use of digital intervention, and use of pharmacotherapy).

Studies that reported biochemically validated abstinence were judged to be at low risk of bias for measurement of the outcome. Seven studies where abstinence was self-reported and not validated biochemically were judged to be at high risk of bias.[50,51,57,63,66,69,79]

The risk of bias from missing outcome data was judged to be low if loss to follow-up was low and similar across arms in the trial. Five studies were judged to be at high risk of bias due to high drop out and/or unequal follow-up between different trial arms.[48,49,57,63,68]

Most studies were judged to be at low risk of bias in selective reporting of results due to adherence to trial protocols and pre-specified sample power calculations.

Overall, six of the 42 included studies were classified as being at low risk of bias on all domains considered in the assessment.[38,52,56,64,71,72]

Five studies were judged to be at high risk of bias due to potential deviations from the intended interventions in the trial. Examples of this include poor participation in or adherence to intervention components (such as behavioural support sessions or use of digital intervention, and use of pharmacotherapy).

Studies that reported biochemically validated abstinence were judged to be at low risk of bias for measurement of the outcome. Seven studies where abstinence was self-reported and not validated biochemically were judged to be at high risk of bias.

The risk of bias from missing outcome data was judged to be low if loss to follow-up was low and similar across arms in the trial. Five studies were judged to be at high risk of bias due to high drop out and/or unequal follow-up between different trial arms.

Most studies were judged to be at low risk of bias in selective reporting of results due to adherence to trial protocols and pre-specified sample power calculations.

Overall, six of the 42 included studies (15%) were classified as being at low risk of bias on all domains considered in the assessment.

Formulas for calculation of pooled effect estimates in random effects meta-analysis

The weight (W_i) of each study in the meta-analysis is given by

$$W_i = 1 / V_i + T^2$$

where V_i is the within-study variance for each study (i) and T^2 it’s between-studies variance, tau-squared*)*.

The weighted effect size X^w is calculated as the sum of the products (effect size *X* multiplied by weight) divided by the sum of the weights

$$X^w = \frac{\sum W_i X}{\sum W_I}$$

The variance v of the pooled effect is given by

$$v = \frac{1}{\sum w_i}$$

and the standard error is computed as the square root of the variance. The 95% confidence intervals for the pooled estimate is then computed as

$$X^w \pm 1.96 \times SE(X^w)$$

Figure s1: Funnel plot of studies included in primary meta-analysis*

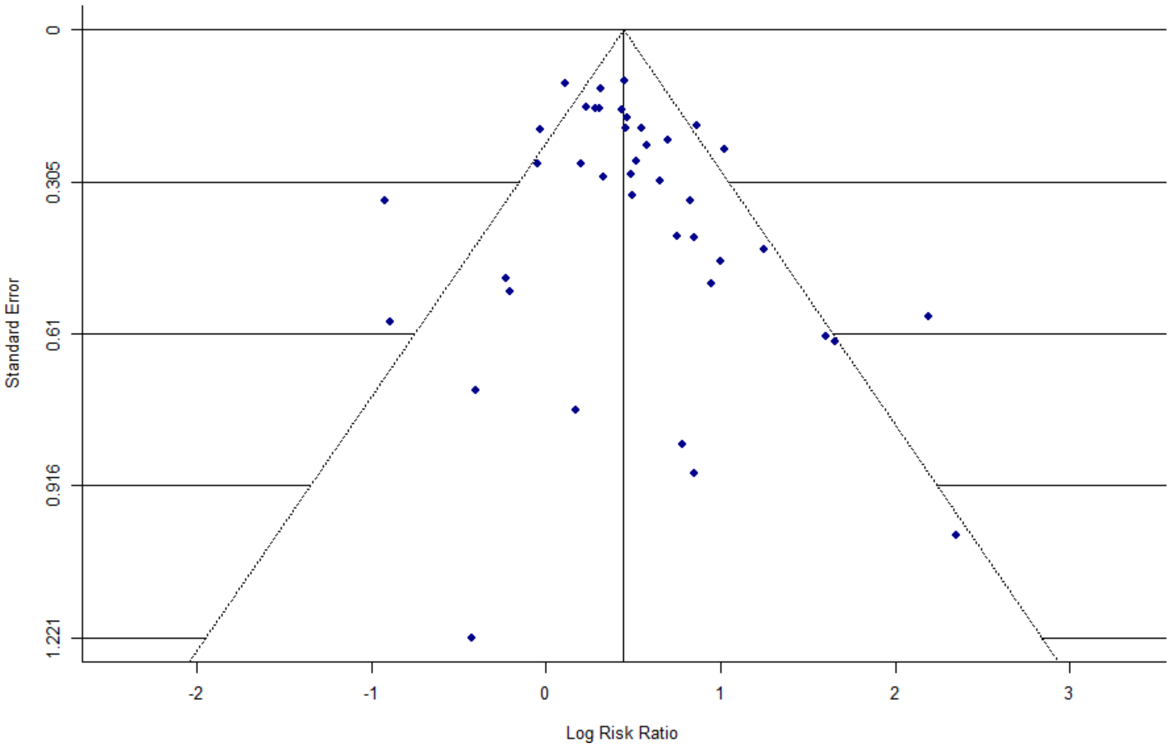

*Standard error of effect size and risk ratio of estimated outcome (smoking cessation)

Table s2: Adjusted associations between intervention factors deemed meaningful and effect size of intervention

Variable	Coefficient *B (SE)*	RR* (95% CI)	*p*
Pharmacotherapy[1]	0.31 (0.11)	1.37 (1.10, 1.71)	.004*
Type of support[2]	-0.37 (0.10)	0.76 (0.62, 0.91)	.004*
Risk of bias[3]	-0.34 (0.13)	0.71 (0.55, 0.93)	.01†

*Risk Ratios (RR) calculated by exponentiating log-transformed estimates (*B)* of intervention effect. Associations after mutual adjustment for all variables listed in Table 4.
Comparisons for binary variables: [1]Pharmacotherapy delivered vs not delivered. [2]Digital or behavioural intervention vs other intervention (financial incentives and brief interventions); [3]High/some concerns risk of bias vs low risk of bias.
†$p < .05$.*$p<.01$

Figure s2: Forest plot of SEP-tailored interventions compared with control/usual care in disadvantaged groups.

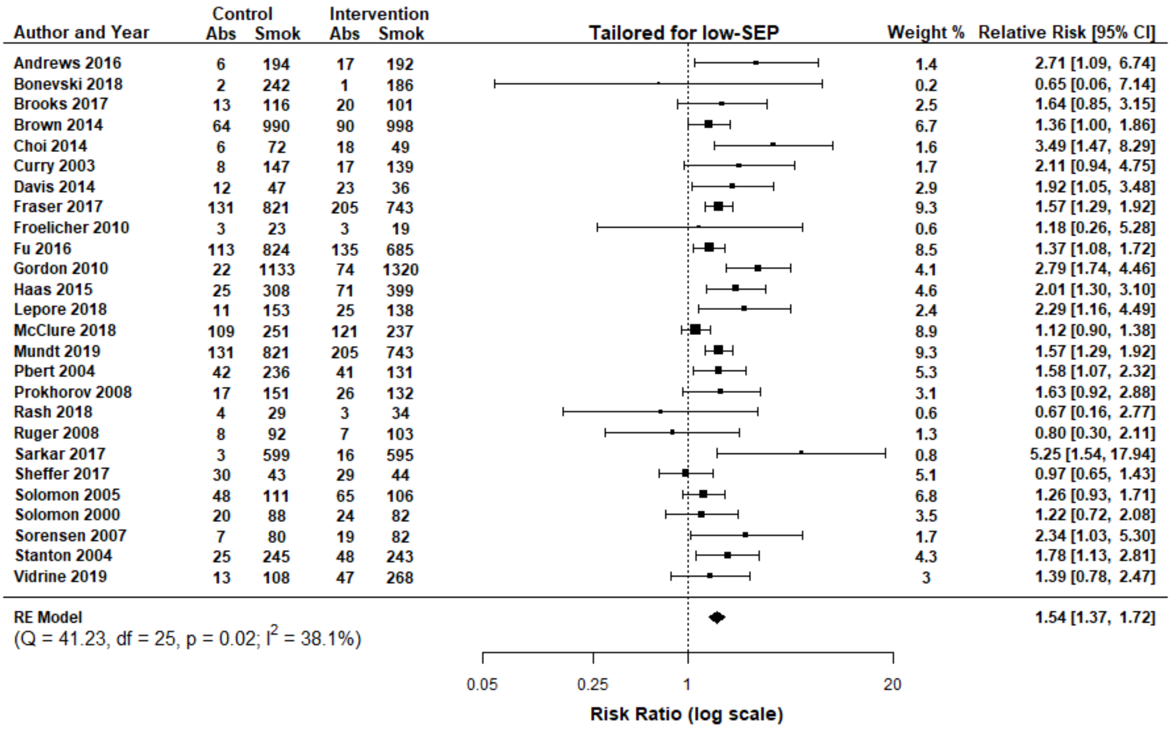

Abs = abstinent; Smok = smoking

Sub-group analysis: comparing non SEP-tailored interventions in low and high SEP participants

To test whether the estimates in each sub-group are different from each other, we fit two separate random-effects models within each subset (low SEP and high SEP) defined by the SEP variable. The SEP variable indicated whether the sample for analysis was 'low' or 'high' SEP.

We then combined the estimates and standard errors from each model into a data frame. A variable was added to distinguish the two models and for reasons explained below, we added the estimated amounts of heterogeneity within each subset to the data frame.

To compare the two estimates (average log risk-ratios), they were fed back into a meta-analysis model using the model variable to distinguish the two estimates as a moderator (highSEP vs lowSEP). A fixed effect model was

used because the residual heterogeneity within each respective subset had already been accounted for by fitting the random-effects models outlined above. The model output is provided below with results in table s3:

Fixed-effects with moderators model

Test for Residual Heterogeneity: QE(df = 0) = 0.000, p-val = 1.000

Test of Moderators (coefficient 2): QM(df = 1) = 0.000, p-val = 0.904

Table s3

	Coefficient *B (SE)*	Z value	*p*	95% CIs
Intercept	0.69 (0.20)	3.54	.001	0.309 - 1.08
LowSEP	-0.03 (0.28)	-0.12	.90	-0.59 - 0.52

The results in table s3 suggest that there are no apparent differences between the estimates of smoking cessation (*B* = -0.03, SE = 0.28, p = .90) according to whether the participants in non SEP-tailored interventions were of ‘low’ or ‘high’ SEP.

Figure s3: Funnel plots of non SEP-tailored interventions (low- and high-SEP participants)*

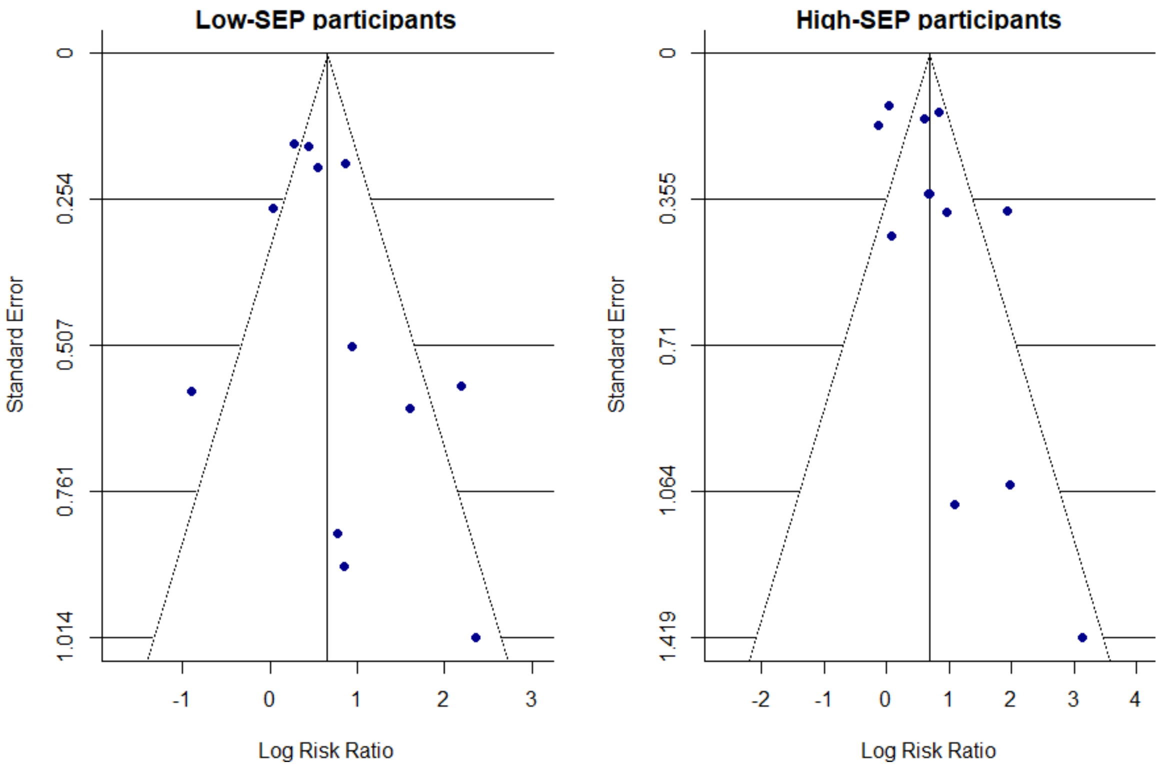

*Standard error of effect size and risk ratio of estimated outcome (smoking cessation)

Egger's regression test

Non SEP-tailored interventions (low-SEP participants)

Regression Test for Funnel Plot Asymmetry

Model: mixed-effects meta-regression model

Predictor: standard error

Test for funnel plot asymmetry: $z = 1.6264$, $p = 0.1039$

Non SEP-tailored interventions (high-SEP participants)

Regression Test for Funnel Plot Asymmetry

Model: mixed-effects meta-regression model

Predictor: standard error

Test for funnel plot asymmetry: $z = 2.3376$, $p = 0.0194$

However, given the high levels of heterogeneity and relatively small number of studies included in the test for asymmetry, the analysis may not be sufficiently powered to distinguish between real asymmetry and chance.

Data extraction reliability

HTB extracted 10% of all study data using the same data extraction form as the primary reviewer (LK). The level of agreement below is calculated based on comparing the data points extracted by LK and HTB for each domain.

Any differences between extracted data were discussed and resolved.

Smoking cessation outcome data (used in intention to treat analysis)

Disagree	1
Agree	51
Total	52
% Agreement	98.1

Trial description data

Disagree	0
Agree	32
Total	32
% Agreement	100

Patient demographics

Disagree	0
Agree	45
Total	45
% Agreement	100

TIDieR

Disagree	4
Agree	230
Total	234
% Agreement	98.3

Pharmacotherapy data

Disagree	0
Agree	24
Total	24
% Agreement	100
